# Retreatment of a Tooth With Calcified Canals Using Cold Ceramic: A Case Report

**DOI:** 10.7759/cureus.90769

**Published:** 2025-08-22

**Authors:** Jalil Modaresi, Mojtaba Mehrabanian, Rita Marincsák, Mina Afshar

**Affiliations:** 1 Endodontics, Shahid Sadoughi University of Medical Sciences, Yazd, IRN; 2 Dentistry, Bupa Dental Care, Dingwall, GBR; 3 Operative Dentistry and Endodontics, University of Debrecen, Debrecen, HUN; 4 School of Dentistry, Shahid Sadoughi University of Medical Sciences, Yazd, IRN

**Keywords:** calcification, chemo-mechanical preparation, cold ceramic, nonsurgical retreatment, periapical lesion

## Abstract

Root canal treatment (RCT) has high success rates; however, failures may occur when chemo-mechanical preparation or obturation are inadequate, leading to persistent infection and periapical lesions. Calcification of the root canal system may obstruct effective canal negotiation and preparation, increasing the risk of under-debridement and treatment failure. When calcified canals cannot be fully managed, the prognosis is typically poor, necessitating extraction or apical surgery. This case report presents nonsurgical retreatment of a symptomatic mandibular left first molar with partially calcified canals. Despite careful negotiation, apical patency could not be safely achieved. Chemo-mechanical debridement with copious irrigations was performed, and the canals were obturated with cold ceramic (CC), a hydraulic calcium silicate-based cement, to establish a corono-apical seal. Symptoms resolved, and radiographs showed near-complete periapical healing maintained at four years. Within the limits of a single case, the favorable outcome is most likely to reflect effective disinfection combined with a secure seal. CC was selected for its biocompatibility, alkaline pH-associated antibacterial effect, and sealing capacity, and it may serve as a pragmatic obturation alternative to gutta-percha when full-length instrumentation is not feasible. A conservative, tooth-preserving approach can be appropriate in similar calcified cases. Further studies are needed to explore CC’s potential and long-term outcomes.

## Introduction

Calcification is a form of root canal obstruction that complicates canal negotiation, impedes achievement of apical patency (gently passing a small file through the foramen [1]) and working length, and increases the risk of inadequate chemo-mechanical preparation, leading to under-obturation and treatment failure [2]. Calcific obliteration has been linked with traumatic injury, orthodontic treatment, caries, abfraction, abrasion, pulp capping, occlusal imbalance, and parafunctional habits. They are more commonly reported in anterior teeth, while less evidence is available for molars [3].

Cases with acute symptoms or apical lesions require endodontic treatment to eliminate microorganisms from the root canal system and prevent reinfection. Predictable success depends on thorough chemo-mechanical preparation of the entire canal system with effective instrumentation and antibacterial irrigation [4]. In some teeth, diffuse calcification completely obstructs the apical third, making negotiation impossible even under magnification with specialized endodontic instruments. In such situations, only the coronal segment to the blockage can be instrumented and sealed, with close radiographic monitoring if the patient becomes asymptomatic. If symptoms persist or recur, or if an apical lesion develops, conventional nonsurgical orthograde retreatment with gutta-percha may have a poor prognosis because the same anatomic constraints prevent full instrumentation and apical control. Although gutta-percha is the most commonly used filling material, it lacks adhesion, has relatively low elastic properties, and can shrink, which may reduce its adaptation to canal walls [5] and result in an inadequate seal [6]. Bioceramic materials have been developed to improve sealing. Mineral trioxide aggregate (MTA) obturation is a promising option for retreatment of challenging nonhealing cases; nevertheless, when MTA cannot reach the apical extent, surgical intervention may be required [6,7].

Cold ceramic (CC) is a hydraulic calcium silicate-based cement containing barium oxide, sulfur trioxide, calcium oxide, and silicon oxide. It is also rich in calcium hydroxide, which enhances alkalinity and antibacterial activity [8,9]. CC shares several benefits with MTA and can improve the MTA's known drawbacks, such as a long setting time, high cost, and potential for discoloration [10,11]. The potential of CC material for endodontic applications has been successfully demonstrated in apexification, perforation repair, and calcified root canals, showing positive effects of CC on bone repair and periapical lesion healing [12-14].

This case report describes nonsurgical retreatment of a symptomatic mandibular first molar with partially calcified canals and a large periapical lesion, using CC as the obturation material.

## Case presentation

**Figure 1:**
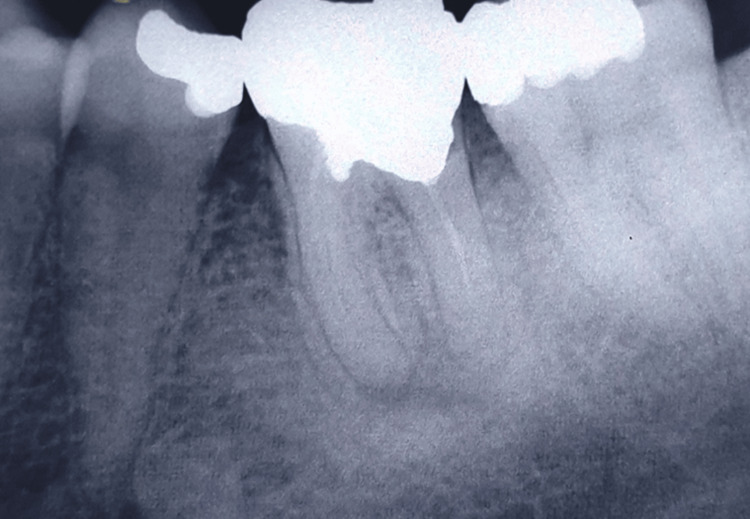
Preoperative radiograph showing incomplete obturation and a large periapical lesion associated with tooth #36

A general dentist referred a 30-year-old man to a private endodontics practice with a chief complaint of pain on biting from a previously endodontically treated mandibular left first molar (#36). The patient had no systemic diseases. The extraoral examination showed normal findings. Intraorally, there were no signs of mobility, traumatic occlusion, visible fracture, or swelling. The periodontal probing depths were ≤3 mm. However, the culprit tooth was sensitive to percussion and palpation. A preoperative periapical radiograph showed an incomplete obturation with a large periapical lesion, bone loss, as well as a partially calcified root canal system associated with tooth #36, as shown in Figure 1. The canals from the prior treatment appeared inadequately instrumented. Unfortunately, the patient did not have access to any radiographs from the primary root canal treatment (RCT).

Differential diagnoses included a cracked tooth and a combined periodontal-endodontic lesion. These were unlikely given normal probing depths without isolated deep pockets, no sinus tract or mobility, a negative bite test for cusp-specific pain, and a periapical radiolucency centered at the root apices consistent with an endodontic source. The working diagnosis was a previously treated tooth with symptomatic periapical periodontitis [1]. After discussing all the treatment options, the patient opted for nonsurgical RCT to save the tooth despite a guarded prognosis. Cone-beam CT (CBCT) was discussed to better delineate canal anatomy and the extent of calcification; the patient declined CBCT due to financial reasons.

Following administration of local anesthesia (1.8 mL of 2% lidocaine with 1:100000 adrenaline) (Darupakhsh Pharmaceutical Co., Tehran, Iran), the retreatment was initiated under rubber dam isolation. After removal of the existing restoration and placement of a pre-endodontic build-up, three orifices were located under a dental operating microscope (DOM) (Zumax® OMS2350, Zumax Medical Co., Ltd., Suzhou, China). The old root canal filling was subsequently removed from the canals with Gates-Glidden drills (Dentsply Maillefer, Ballaigues, Switzerland) and rotary files (FKG Dentaire, Le Crêt-du-Locle, Switzerland). C-files size #10 (VDW GmbH, Munich, Germany) with chloroform (Merck KGaA, Darmstadt, Germany) were used to initially negotiate the canals and reach the appropriate working length.

Despite extended efforts, full working length was not achieved due to the obstruction by severe calcification in the apical third of the canals. The tooth prognosis was deemed hopeless. All possible treatment options, including endodontic surgery or extraction, were discussed again with the patient, who elected to proceed with a tooth-preserving attempt via nonsurgical retreatment.

**Figure 2:**
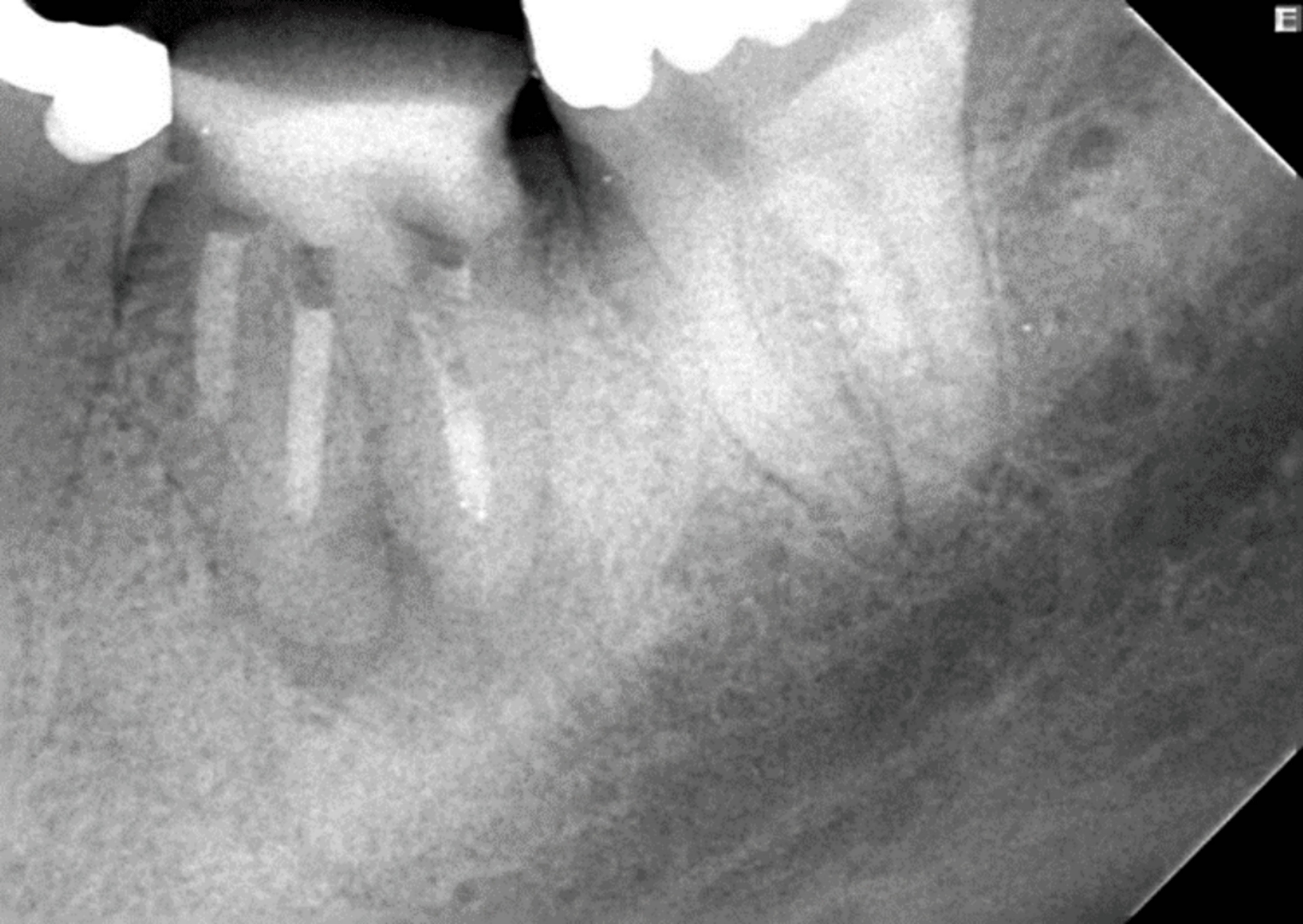
Immediate postoperative mesioexcentric radiograph showing obturation of the root canal system with cold ceramic

Accordingly, the three canals were chemomechanically debrided within the accessible segments up to the points of obstruction. Cleaning and shaping of the root canal were performed with K-file #70 hand file (Mani, Tochigi, Japan) using 5% sodium hypochlorite (NaOCl), 17% ethylenediaminetetraacetic acid (EDTA), and saline as irrigants. A NaOCl-EDTA-NaOCl irrigation sequence was used with normal saline in between and as a final rinse before obturation. All solutions except saline were ultrasonically activated for 20 seconds using the Woodpecker Ultrasonic UDS-E LED unit with an E2 tip (Guilin Woodpecker Medical Instrument Co., Ltd., Guilin, China). Once the canals were prepared chemo-mechanically, dried, and free of exudate, retreatment was completed in a single visit, and obturation was performed in the same session orthogradely with CC (Samin SJM Co., Yazd, Iran) according to the manufacturer’s instructions. CC was delivered with an MTA carrier (Angelus, Londrina, Paraná, Brazil) and then condensed using Plugger #25 (Dentsply Maillefer, Ballaigues, Switzerland). Efforts were made to prevent any porosities in the CC packing. The adequate space for the placement of a post in the distal canal was reserved during packing. After obturation, a sterile polytetrafluoroethylene (PTFE) tape barrier (3M ESPE, St. Paul, USA) was placed over the canal orifices/CC, and the access was temporized with Cavit (3M ESPE, St. Paul, USA) to provide a coronal seal. Occlusion was adjusted as required, and the patient was dismissed. Figure 2 shows the immediate postoperative radiograph, indicating partially calcified canals filled with CC to the level of calcification and the existing large periapical lesion. For symptom control, the patient was advised to use over-the-counter analgesics as required; antibiotics were not prescribed because there were no systemic signs or signs of spreading infection.

**Figure 3:**
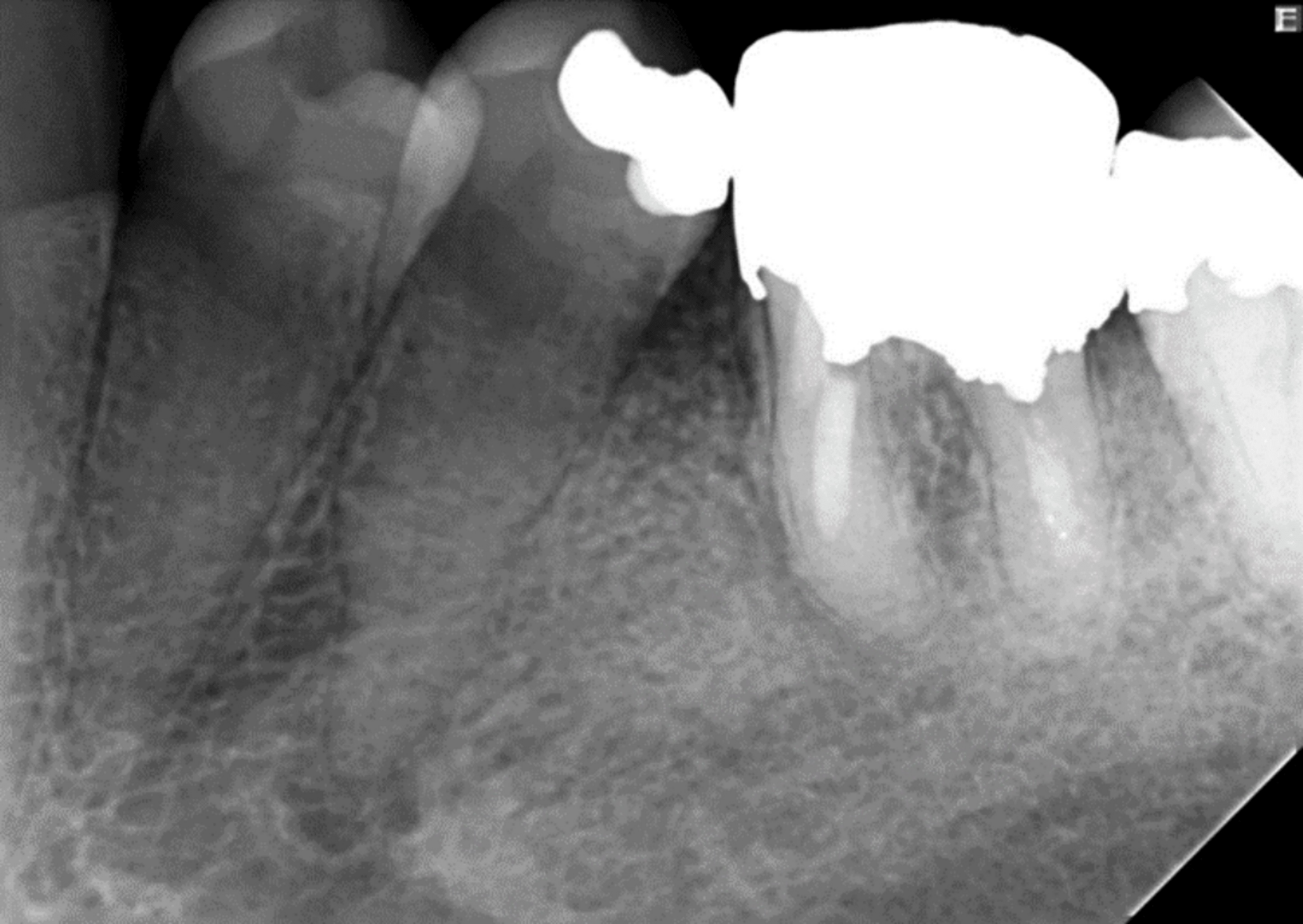
Four-year follow-up radiograph showing periapical bone regeneration and a normal periodontal ligament

One week later, the patient reported pain resolution by day 3 after treatment. No analgesics were used after day 3, and normal mastication returned within one week. No swelling, paresthesia, or flare-ups were reported at any time. The asymptomatic patient was referred back to his dentist for definitive coronal restoration; a full-coverage crown with distal post placement was recommended. Scheduled endodontic follow-up was clearly advised, but the patient did not attend interim reviews. Four years later, he re-presented for unrelated care and admitted continued absence of symptoms with satisfactory function and aesthetics of the lower first molar. A review radiograph showed near-complete healing of the periapical radiolucency with re-establishment of the periodontal ligament around the apices of both mesial and distal roots (Figure 3). Clinically, mobility was physiologic (Miller Grade 0), probing depths were ≤3 mm, and function was normal without symptoms. Although secondary caries was absent, an overhanging restoration was present, and the patient was referred to his dentist for correction.

## Discussion

Successful RCT depends primarily on microbial load control achieved by optimal chemo-mechanical preparation, followed by prevention of reinfection through a reliable apical and coronal seal [15]. In severely calcified canals, achieving apical patency and full working length may be unsafe or impossible; in such scenarios, the priority is to disinfect the accessible canal space and establish a durable seal while avoiding iatrogenic damage.

In this case, severe apical-third calcification prevented safe negotiation to patency. High-volume irrigation with 5% NaOCl and 17% EDTA, supported by ultrasonic activation, was used to debride the accessible segments. The healing at four years despite the absence of patency supports the view that effective chemical disinfection rather than maximal mechanical enlargement is one of the principal determinants of success [6,16]. Irrigants help create an aseptic environment, dissolve and flush necrotic tissue, and neutralize bacterial toxins in hard-to-reach areas [16].

Moreover, long-term success also requires prevention of reinfection, which can be ensured by a fluid-tight seal [6]. CC was selected as a hydraulic calcium silicate cement with biocompatibility, alkaline pH-associated antibacterial activity, and sealing/adaptation potential. These properties may have contributed to preserving the aseptic environment created by irrigation and to limiting microleakage [8,9]. Furthermore, CC cannot be used as a filling material in all cases due to its irretrievability. However, we used it as a last resort to potentially avoid surgery or tooth removal and subsequent implant or bridge placement.

Limitations include the absence of CBCT (declined by the patient), reliance on 2D radiographs for assessing anatomy and calcification, and lack of baseline images from the primary RCT. The patient missed interim reviews, so patient-reported outcomes between one week and four years were not captured. CC’s irretrievability is a practical constraint. As a single-patient report without a control group, the findings are not generalizable and should be interpreted cautiously.

## Conclusions

Within the limits of a single case, periapical healing was achieved despite the inability to obtain apical patency when effective chemo-mechanical disinfection was combined with an immediate corono-apical seal using CC as a pragmatic obturation alternative to maintain the disinfected state when full-length instrumentation was not feasible. The favorable outcome most likely reflects adequate disinfection, which resulted in a low residual microbial load, with CC acting as a contributory sealing material that enabled a conservative, tooth-preserving approach and avoided endodontic surgery or extraction.
